# Prevalence of Diabetes Mellitus in Patients with Chronic Kidney Disease

**DOI:** 10.3889/oamjms.2016.019

**Published:** 2016-01-20

**Authors:** Olivera Stojceva-Taneva, Natasa Eftimovska Otovic, Borjanka Taneva

**Affiliations:** 1 *University Clinic for Nephrology, Medical Faculty, Ss Cyril and Methodius University of Skopje, Skopje, Republic of Macedonia*; 2 *City General Hospital “8th of September”, Medical Faculty, Ss Cyril and Methodius University of Skopje, Skopje, Republic of Macedonia*; 2 *University Clinic for Cardiology, Medical Faculty, Ss Cyril and Methodius University of Skopje, Skopje, Republic of Macedonia*

**Keywords:** chronic kidney disease, diabetes mellitus, risk factors, prevalence

## Abstract

**BACKGROUND:**

Chronic kidney disease (CKD) became a new epidemic of the twentieth and twenty-first centuries. Diabetic nephropathy is one of the leading causes of end-stage renal failure as a result of the diabetes epidemic worldwide.

**AIM:**

The aim of our study was to assess the prevalence of CKD in the Republic of Macedonia and its association with diabetes mellitus.

**MATERIALS AND METHODS:**

The study was a part of a study conducted in 2006 in terms of screening for early detection of kidney disease. It was a cross-sectional study based on a random sample of patients aged > 20, consecutively consulting their primary physician for any cause. Fifty physicians throughout the country were included in the study. A total of 2637 patients have been analyzed based on integrity data. GFR was estimated using corrected values of serum creatinine and calculating kidney function by the Cockroft & Gault formula, adjusted for body surface using the Gehan & George formula. Patients with estimated glomerular filtration rate (eGFR) less than 60 ml/min were considered as having CKD. Blood pressure, body weight, height, serum creatinine, glucose, hemoglobin, hematocrit, urinalysis and medical history for presence of cardiovascular diseases or diabetes were also assessed.

**RESULTS:**

The mean age of the subjects was 45.97 ± 16.55 SD and 17.97% were older than 60. Regarding gender, 44.14% were males. The prevalence of diabetes mellitus was 13.9%. Subjects with CKD (eGFR less than 60 ml/min) were 7.53% of the total. Subjects aged 60 or above, had 20 times higher risk of having CKD (eGFR less than 60 ml/min/1.73 m^2^). Out of the total group of subjects, 13.9% had diabetes mellitus and they had 3.13 times higher risk of having CKD stage 3-5 (eGFR less than 60 ml/min/1.73 m^2^) when compared to non-diabetics. The results showed that diabetes was significantly more associated with lower eGFR (less than 60 ml/min/1.73 m^2^) in younger subjects (age less than 60) compared to older ones (odds ratio 3.29 versus 1.21).

**CONCLUSION:**

Our study showed that chronic kidney disease is frequent in the Republic of Macedonia and is associated with older age and diabetes. Diabetes had a significantly stronger association with CKD at younger age.

## Introduction

Chronic kidney disease (CKD) became a new epidemic of the twentieth and twenty-first centuries. At present, it is a global problem, mainly because a variety of risk factors are being involved in its etiology and pathophysiology. It is not surprising that diabetic nephropathy is one of the leading causes of end-stage renal failure having in mind the diabetes epidemic worldwide [1].

The prevalence of CKD differs among countries and ethnicities. It is of great importance for the policy makers to know the prevalence of CKD, as it is associated with high morbidity and mortality and high cost of renal replacement therapy. Guidelines from the National Kidney Foundation’s Kidney Disease Outcomes Quality Initiative (KDOQI) recommend estimating glomerular filtration rate and screening for albuminuria in patients with risk factors for chronic kidney disease, including diabetes, hypertension, systemic illnesses, age greater than 60 years, and family history of chronic kidney disease. The glomerular filtration rate (GFR), calculated by using a prediction equation, detects chronic kidney disease more accurately than does the serum creatinine level alone; the GFR also is used for disease staging. Current KDOQI guidelines recommend screening for kidney disease with a serum creatinine measurement for use in GFR estimation and analysis of a random urine sample for albuminuria [2].

The prevalence of CKD has increased dramatically in the period of 1999 to 2007 in different countries around the world, developed, as well as developing countries. It has reached epidemic proportions with 10–13% of the populations in Taiwan, Iran, Japan, China, Canada, India and the USA [3].

The aim of this paper was to estimate the prevalence of CKD in the Republic of Macedonia and the association of diabetes mellitus in patients with CKD not on renal replacement therapy.

## Materials and Methods

This study is part of a study conducted in August/September 2006 in terms of screening for early detection of kidney disease (SKROBB). It was a cross-sectional study based on a random sample of patients aged > 20, consecutively consulting their primary physician for any cause. Fifty physicians throughout the country were included in the study. A total of 3019 patients were included, but 2637 have been analyzed based on integrity data. The physicians were asked to fill in a questionnaire for demographic and medical history data. They were also required to measure blood pressure, body weight, height, serum creatinine, glucose, hemoglobin, hematocrit and urinalysis. Proteinuria (qualitatively, 1+ or more), and/or hematuria, and estimated glomerular filtration rate (eGFR) < 60 ml/min per 1.73 m^2^ have been considered as markers of renal damage or of chronic kidney disease. Serum creatinine has been measured by the use of Jaffe’s method, and in order to minimize the differences among alkaline picrate methods regarding influence of protein on creatinine results, at least in 10% of all the samples of each laboratory creatinine has been measured at the reference laboratory at the Medical Faculty in Skopje. A correction factor for creatinine has been estimated in each laboratory in the country. The body surface area has been measured using the Gehan & George formula [4]. The eGFR has been estimated using the Cockroft & Gault formula (Calculated creatinine clearance = [(140-age) x 1.23 x BW(kg)/Scr] x 0.85 if female). Gender, age, presence of cardiovascular diseases, hypertension, diabetes, anemia, and obesity (BMI) has been considered as risk factors for CKD. Hypertension was defined as systolic blood pressure (SBP) >140 mmHg and diastolic blood pressure > 90 mmHg or having medical history or being treated for hypertension [5]. Persons were considered diabetic if fasting blood glucose was more than 7.1 mmol/l, or having medical history or being treated for diabetes [6].

The prevalence of CKD was calculated after adjustment for age and gender to the general population (the population census in 2002 was taken; the total population aged > 20 was 1,429,642).

Stage 1 and 2 CKD was considered on the basis of proteins in the urine (1+ or more) without evidence of urinary infection.

Statistical analysis has been conducted using the software program Statistica for Windows 10.0. Student-t test was used to compare the numerical data. Mantel-Haenszel Chi-square test was used to see the relationship between two dichotomous variables. A p-value < 0.05 has been taken as statistically significant.

## Results

The study included 2,637 subjects based on integrity data. The mean age of the subjects was 45.97 ± 16.55 SD and 17.97% were older than 60. Regarding gender, 44.14% were males. The relevant demographic and clinical data were the following: prevalence of diabetes mellitus was 13.9%, hypertension had 36.37% of subjects, actual smokers were 28.02%%, previous smokers and non-smokers were 12.49% and 59.08% respectively. According to ethnicity, Macedonians were 74.42%, Albanians 17.23%, Roma 5.67% and others 2.68%. Anemia (hemoglobin less than 110 g/l) had 8.52% of subjects, history of cardiovascular diseases 37.46%, subjects living in urban area were 83.63%, and rural 14.42 %.

Table 1 shows the prevalence of CKD stages in the investigated population adjusted by age and gender to the general population. The population census in 2002 has been used. Stages 1 and 2 were defined by presence of proteins in the urine (1+ or more).

**Table 1:** Prevalence of CKD stages estimated by Cockroft-Gault (adjusted by age/gender)

CKD stage	% male	Per million males	% females	Per million females	% total	Per million total
Stage 1	3.97	39.758.00	3.2	32.445.00	3.57	35.673.4
Stage 2	2.11	21.132.2	3.51	35.129.8	2.89	30.103.0
Stage 3	5.5	54.969.7	8.4	84.357.0	7.1	71.384.9
Stage 4	0.24	2385.5	0.27	2677.6	0.25	2548.6
Stage 5	0.07	717.4	0.26	2622.9	0.18	1781.8
Total					**13.99**	

Table 2 shows that subjects older than 60 years have a 20 times greater risk of having CKD (eGFR less than 60 ml/min/1.73 m^2^) compared to those being less than 60 years of age.

**Table 2:** Association of age and low eGFR

Age	eGFR ≥ 60 ml/min/1.73m^2^	eGFR < 60 ml/min/1.73m^2^
≥ 60 years	395	175
< 60 year	2008	43
Total	2403	218

Mantel-Haenszel = 478.43; (95% CI: 14.6-29.4); OR = 20.7; p < 0.001.

Table 3 shows the presence of diabetes in different levels of eGFR. Those having established CKD, eGFR less than 60 ml/min/1.73 m^2^ have higher percentage of diabetics compared to those with eGFR above 60 ml/min/1.73 m^2^.

**Table 3:** Presence of diabetes mellitus (DM) in subjects according to the level of eGFR

Egfr Ml/Min/1.73m^2^	Presence of DM	Absence of DM	Total	No Data	Total	%
≥90	126	1431	1557	30	1587	7.9%
60-89	166	632	798	18	816	20.3%
30-59	64	140	204	3	207	30.9%
15-29	2	5	7	0	7	28.6%
<15	0	4	4	0	4	0
Total	358 (13.9%)	2212	2570	51	2621	

Table 4 shows that those having diabetes have 3 times higher risk of having CKD (eGFR less than ml/min/1.73 m^2^) compared to those who do not have diabetes.

**Table 4:** Association of diabetes and lower eGFR (less than 60 ml/min/1.73 m^2^)

Presence of DM	GFR ≥60 ml/min/1.73 m^2^	GFR <60 ml/min/1.73 m^2^
Yes	292 (81.6%)	66 (18.4%)

No	2063 (93.3%)	149 (6.7%)

Mantel-Haenszel = 54.9 (95% CI 2.28-4.28), OR- 3.13 p < 0.001.

After correction for age, it appeared that subjects aged less than 60 and having diabetes, have greater risk for having lower eGFR (less than 60 ml/min/1.73 m^2^) compared to subjects with diabetes that are older than 60 (Table 5). It indicates that diabetes is significantly more associated with lower eGFR in younger subjects. Age is a confounding variable. In order to see whether age (below/above 60) will have an impact upon a subject with diabetes to have lower eGFR (less than 60 ml/min/1.73 m^2^), a statistical analysis using 3 x 2 table (2 x 2 x 2) was performed, and it showed a Chi-square of 5.991, degrees of freedom 1, and p = 0.0144. This result confirms that the difference in odds ratios between 3.29 and 1.1 is significant. Thus, we can conclude that age significantly predisposes the subject with diabetes towards having lower eGFR.

**Table 5:** Association of diabetes with lower eGFR after correction by age

DM	Age < 60		Age ≥ 60	

	GFR ≥ 60	GFR < 60	GFR > 60	GFR < 60
Yes	172 (94.5%)	10 (5.5%)	120 (68.2%)	56 (31.8%)

No	1757 (98.3%)	31 (1.73%)	306 (72.2%)	118 (27.8%)

For age less than 60: For age above 60: Mantel-Haenszel – 11.458, p < 0,001 Mantel-Haenszel 0.959; p = 0.3274; 95% CI 1.58-6.83; 95% CI 0.826-1.77; OR-3.29; OR-1.21.

## Discussion

Data on the epidemiology of CKD in predialysis stages exists only in a small minority of countries in Europe. So, there is lack of information on prevalence of CKD in the different stages in different countries [7]. The available studies from the USA, Europe, Australia, and Asia showed that the prevalence of CKD is about 9–13% in the general population. The incidence and prevalence of patients with CKD including end-stage renal disease (ESRD) have doubled in the past 10 years in the USA and it is increasing not only among adults in the United States but also worldwide [8-10]. The incidence and prevalence of CKD increase markedly at older age [9, 11]. For example, in the Framingham Heart Study, the risk of developing stage 3 CKD was 2.36 times higher for each 10 years older age [11]. Additionally, the prevalence of stage 1 or 2 and stage 3 or 4 CKD among US adults has been reported to be 3.3 and 54 times higher, respectively, for adults ≥70 versus 20 to 39 years of age [9]. Our study also showed a strong correlation of age and CKD (20 times greater risk of having CKD if a subject is 60 years of age or older).

In the study of Islam T. et al, for adults 20 to 49, 50 to 69 and ≥ 70 years of age, the prevalence ratios (95% confidence interval) of stage 3 or 4 CKD associated with diagnosed diabetes mellitus were 3.01 (1.35 – 6.74), 1.61 (1.15 – 2.25), 1.40 (1.15 – 1.69), respectively, p-trend = 0.067; and 2.67 (0.53 – 13.4), 1.35 (0.69 – 2.63), 1.08 (0.78 – 1.51), respectively, for undiagnosed diabetes mellitus (p-trend = 0.369) [10]. Our study also showed strong correlation of diabetes mellitus and CKD, Subjects had 3 times higher risk of having CKD stage 3-5 if being a diabetic. Age in diabetic subjects was a confounding variable for having CKD in our study. Subjects with DM and older than 60 had 1.2 higher risk of having CKD, compared to those with DM and younger than 60 (who had 3.3 times higher risk of having CKD). In the study of Islam [12], although not statistically significant, there was also a trend towards lower prevalence ratios of stage 3 or 4 CKD at older age for diagnosed (p-trend = 0.067) and undiagnosed diabetes mellitus (p-trend = 0.369).

This study has two limitations. The study population were patients consecutively consulting their general physician for any reason, and not subjects from the general population. Therefore, a possible selection bias must be considered. The high number of patients with diabetes mellitus in the study population points out to this notion. Secondly, stage 1 and 2 CKD were defined by qualitative measurement of protein in the urine, and not by albuminuria or albumin/creatinine ratio. Therefore, the presence of CKD stage1 and 2 may not be completely accurate. Hence, to show the association of age and diabetes with CKD, we take into consideration only CKD stage 3-5 (eGFR less than 60 ml/min/1.73 m^2^).

In conclusion, our study showed that chronic kidney disease is frequent in the Republic of Macedonia and is associated with risk factors as older age and diabetes. Diabetes mellitus is associated with stage 3 to 5 CKD across the full adult age range. But, this association was statistically significantly stronger at younger age.
